# An atmospheric origin of the multi-decadal bipolar seesaw

**DOI:** 10.1038/srep08909

**Published:** 2015-03-10

**Authors:** Zhaomin Wang, Xiangdong Zhang, Zhaoyong Guan, Bo Sun, Xin Yang, Chengyan Liu

**Affiliations:** 1 Polar Climate System and Global Change Laboratory, Nanjing University of Information Science and Technology, Nanjing, China. 210044; 2 Earth System Modelling Center (ESMC), Nanjing International Academy of Meteorological Sciences(NIAMS), Nanjing University of Information Science and Technology, Nanjing, China. 210044; 3 International Arctic Research Center and Department of Atmospheric Sciences, University of Alaska Fairbanks, 930 Koyukuk Dr., Fairbanks, Alaska 99775, USA; 4 Polar Research Institute of China, No. 451 Jinqiao Road, Pudong District, Shanghai. 200136, China; 5 British Antarctic Survey, High Cross, Madingley Road, Cambridge CB3 0ET, UK

## Abstract

A prominent feature of recent climatic change is the strong Arctic surface warming that is contemporaneous with broad cooling over much of Antarctica and the Southern Ocean. Longer global surface temperature observations suggest that this contrasting pole-to-pole change could be a manifestation of a multi-decadal interhemispheric or bipolar seesaw pattern, which is well correlated with the North Atlantic sea surface temperature variability, and thus generally hypothesized to originate from Atlantic meridional overturning circulation oscillations. Here, we show that there is an atmospheric origin for this seesaw pattern. The results indicate that the Southern Ocean surface cooling (warming) associated with the seesaw pattern is attributable to the strengthening (weakening) of the Southern Hemisphere westerlies, which can be traced to Northern Hemisphere and tropical tropospheric warming (cooling). Antarctic ozone depletion has been suggested to be an important driving force behind the recently observed increase in the Southern Hemisphere's summer westerly winds; our results imply that Northern Hemisphere and tropical warming may have played a triggering role at an stage earlier than the first detectable Antarctic ozone depletion, and enhanced Antarctic ozone depletion through decreasing the lower stratospheric temperature.

Data from ship-based hydrographical surveys and autonomous floats have shown a substantial warming in the Southern Ocean (SO) from the 1930s to the 1970s, but little change thereafter^1^. Further analysis of longer-term global sea surface temperature (SST)^2^ and polar surface air temperature (SAT)^3^ suggest multi-decadal interhemispheric seesaw (or “bipolar seesaw”) patterns that are well correlated with the multi-decadal oscillations of North Atlantic SST^2^,^3^, termed the Atlantic Multi-decadal Oscillation (AMO). It has been generally hypothesized, therefore, that multi-decadal oscillations of the Atlantic Meridional Overturning Circulation (AMOC) could be a cause of this fluctuating bipolar seesaw (e.g., refs. 2 and 3), as the AMOC connects both poles because it transports heat northward in both hemispheres. When AMOC strengthens, northward oceanic heat flux increases, warming the Northern Hemisphere (NH) but cooling the Southern Hemisphere (SH) (e.g., see ref. 4). The recent phase of the interhemispheric or bipolar seesaw, including warming in the northern North Atlantic and comparative cooling in the SO, has led to the speculation that AMOC has been strengthening since the 1970s^5^.

However, recent reconstructions suggested a weakening of the AMOC since the 1970s (e.g., ref. 6). Results derived from current surface heat flux data and observed heat content in the SO also do not support a strengthening of the AMOC since the 1970s (see Supplementary Section I). These suggest that the atmospheric component of the coupled air-sea interactive system may play a contributing role in the formation of the multi-decadal interhemispheric or bipolar seesaw, which has not been well considered in previous studies (e.g., refs. 2, 3, and 5). We therefore conducted a synthetic analysis to untangle the responsible atmospheric processes. We will first evaluate the robustness of this seesaw pattern, in particular after 1950 when the fidelity of observational data was enhanced, then analyze the best available data to identify an atmospheric origin of the multi-decadal interhemispheric or bipolar seesaw in surface temperature, and finally elucidate the mechanism by which it may operate. Our results will thus reveal dynamical consistency among different long-term observational data sets, at least for the time period after 1950.

## Results

### Robustness of the multi-decadal bipolar seesaw after 1950

**Figure 1:**
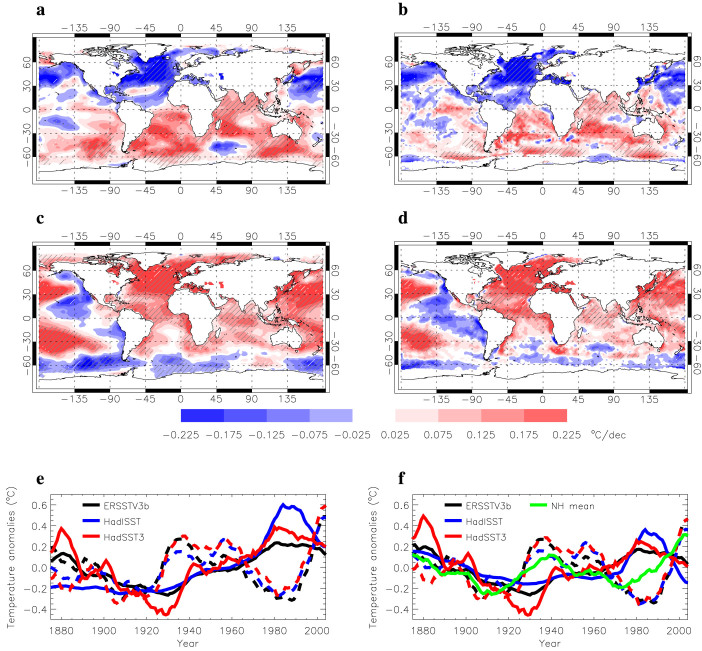
Contrasting interhemispheric and bipolar surface temperature changes. a, b, c, d, Linear trends in ERSSTV3b SST between 1950 and 1979 (a); HadISST SST between 1950 and 1979 (b); ERSSTV3b between 1980 and 2009 (c); HadISST SST between 1980 and 2009 (d); Significance levels indicated by hatched areas are less than 5%. e, f, time series of SST anomalies (e) and detrended SST anomalies relative to their long-term means (f). In e and f, time series are smoothed by applying the 11-year running mean (black lines: ERSSTV3b; blue lines: HadISST SST; red lines: HadSST3; solid lines: SSTs averaged over 70°S–50°S, the SO SST; dashed lines: the SSTs averaged over 50°N–70°N, the Atlantic sector, northern SST.) In f, the correlation coefficients between the SO SSTs and northern SST after 1950 are −0.50 (p < 0.01) for ERSSTV3b, −0.84 (p < 0.01) for HadISST, and −0.74 (p < 0.01) for HadSST3, and the lagged correlation coefficients between the SO SSTs and the NH mean surface temperature (green line) (with the latter leading the former by 10 years) are 0.69 (p < 0.01) for ERSSTV3b, 0.80 (p < 0.01) for HadISST, and 0.85 (p < 0.01) for HadSST3; the significance levels of the correlations are determined by using the non-parametric random phase method^10^. Note that using 21- and 31-year running mean to smooth the time series do not change the correlation coefficients and significance levels significantly. This figure was plotted by using Interactive Data Language.

To enhance credibility in our examination of interhemispheric or bipolar seesaw robustness, we employed two state-of-the-art compilations of SST data, including the Extended Reconstructed SST v3b^7^ (ERSSTV3b; no satellite data are used in this version) and the Hadley Centre Global SST (HadISST) data^8^ to derive decadal-scale linear trends in annual mean global SSTs. Because of the dramatically improved SST data quality after the mid-20^th^ century (Supplementary Section II), we focused on the latest time period of 1950–2009. The linear trends in annual mean global SSTs over the two sub-time periods of 1950–1979 and 1980–2009 highlight contrasting changes between the SH and NH (Fig. 1a–d). During 1950–1979, the NH exhibited cooling and the SH warming; during 1980–2009, opposite changes occurred at high latitudes of both hemispheres, though broader warming extended into the SH due to a superimposed global-warming-forced long-term trend (hereafter we use the term “bipolar seesaw” to refer to this contrasting interhemispheric or bipolar surface temperature change).

The time series of the SO SST (averaged SST over 70°S–50°S) and northern SST (averaged SST over 50°N–70°N, the Atlantic sector) derived from the recently updated (but uninterpolated) SST compilation, HadSST3^9^, along with those derived from ERSSTV3b and HadISST, further illustrate the enhanced robustness of this bipolar seesaw after 1950 (Fig. 1e–f). The correlation coefficients between the detrended SO SSTs and northern SSTs in Fig. 1f after 1950 are −0.74 for HadSST3, −0.50 for ERSSTV3b, and −0.84 for HadISST; significance levels were determined to be less than 1% using the non-parametric random phase method^10^. The NH mean surface temperature (http://www.cru.uea.ac.uk/cru/data/temperature/) is also well correlated with the SO SSTs with a lead of about 10 years (Correlation coefficients are −0.85 for HadSST3, −0.69 for ERSSTV3b, and −0.80 for HadISST, with the NH mean surface temperature leading the SO SSTs by 10 years, and their significance levels are less than 1%.). The lagged SO SST changes are presumably induced by deep convection at southern high latitudes.

While ref. 11 questioned the existence of the bipolar seesaw in polar SAT found in ref. 3 by considering the sparsity of historical Antarctic station data, the results from analyzing the state-of-the-art SST datasets in this study demonstrate that the bipolar seesaw pattern is a robust feature at least after 1950 when instrumental data quality dramatically improved (see Supplementary Sections II and III for more robustness analysis). This robust feature offers an observational basis for the mechanism analysis below.

### Relationship between the SO SST variability and the Southern Annular Mode

To explore atmospheric impacts upon the bipolar seesaw, we examined the relationship between SO SST variability and the Southern Annular Mode (SAM)^12^, the leading atmospheric circulation mode over southern high latitudes. An increase (decrease) of the SAM index indicates a strengthening (weakening) and poleward (equatorward) shift in the SH westerly jet.

**Figure 2:**
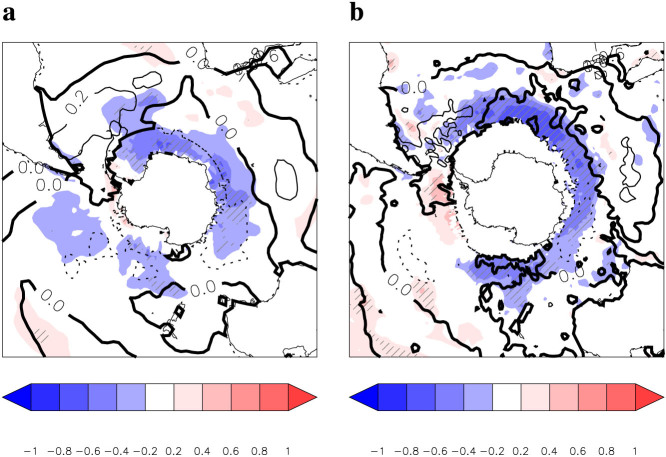
SO SST changes driven by SAM. a, b, Linear regressions of annual mean ERSSTV3b SST (a) and HadISST SST (b) on the Visbeck SAM index (colour filling) for the period of 1884 to 2005, which are similar to the results for the period of 1950 to 2005. Time series are smoothed using the 11-year running mean, detrended, and normalized. Significance levels indicated by hatched areas are less than 5%. For a comparison with the SAM-SST relationship on a seasonal time scale that may not be affected by slower oceanic processes, the annual means of the monthly regressions of the SST on the SAM for the 1981–2005 period are shown as contour lines (Negative contours are dashed, and the zero contour is highlighted in bold; contour interval: 0.2). This figure was plotted by using Interactive Data Language.

The long-term SAM index back to 1884 was derived by using mid-latitude and Antarctic station surface pressure data or by the concept of atmospheric mass conservation poleward of 20°S when Antarctic station data were not available before the Second World War (for details see ref. 12; this SAM index is consistent with other SAM index reconstructions, as demonstrated in Supplementary Section V and Fig. S5). A regression analysis of annual mean SST on the SAM index^12^ shows a general cooling pattern over the SO, except for a slight warming around the Antarctic Peninsula and the Bellingshausen Sea (Fig. 2), corresponding to an increase in the SAM. This pattern is broadly consistent between the two SST datasets, though some regional differences exist, e.g., to the west of the Drake Passage, presumably due to sparse direct observational data in these regions and different approaches used to construct the gridded data sets. The roles of various physical processes associated with the strengthening of westerly winds, including Ekman drift, cloud reflection of shortwave radiation, and oceanic heat transports, in forcing the multi-decadal SO SST change have been examined (e.g., refs. 13,14,15,16). Our results here suggest that the strengthening of westerly winds may exert a net cooling effect on SO SST., i.e., that the associated cooling effects of those processes, including enhanced cold northward Ekman drift^13^ and increased cloud reflection of short wave radiation^14^, dominate over the warming effects induced by the poleward shift of the Antarctic Circumpolar Current^15^ and increased poleward eddy heat transport^16^ when westerly winds strengthen.

### Driving role of NH and tropical thermal states in the SAM variability

What mechanisms are responsible for the observed changes in SAM? Extensive studies have demonstrated that both Antarctic ozone depletion and global warming could have driven the strengthening of the SH westerly winds or the increasing SAM index since the 1970s (e.g., refs. 17,18,19, among others); in particular, Antarctic ozone depletion may have a much larger impact on the SH tropospheric circulation in summer than in other seasons through cooling the lower stratosphere^18^. Because the first detectable Antarctic ozone decline occurred in the late 1970s^20^ and a large ozone hole began forming in 1982^21^, we extended the study presented here to a longer period to examine how global mean temperature changes may impact SAM in the context of the bipolar patterns. Our study, therefore, includes the periods with both decreasing and increasing SAM phases and with and without an Antarctic ozone hole. Examining the relationship between SAM and global mean temperature over a period without an Antarctic ozone hole facilitates identifying the role of global temperature change in driving SAM variability.

Contributions to global mean temperature changes are strongly dependent on geographic regions. Due to much larger land cover and hence much larger surface temperature changes in the NH than in the SH, NH mean surface temperature change dominates global mean surface temperature change. Also, the averaged surface temperatures over the NH land mass and the North Atlantic, two relatively well-observed regions, are highly correlated with NH and hence global temperature changes. Therefore, to examine the impact of global atmospheric temperature change on SAM multi-decadal variability, we first used the surface temperature variability or change that occurred over the NH, the NH land mass^22^, and the North Atlantic (over 0–70°N; average SST is used to define the AMO index after detrending).

**Figure 3:**
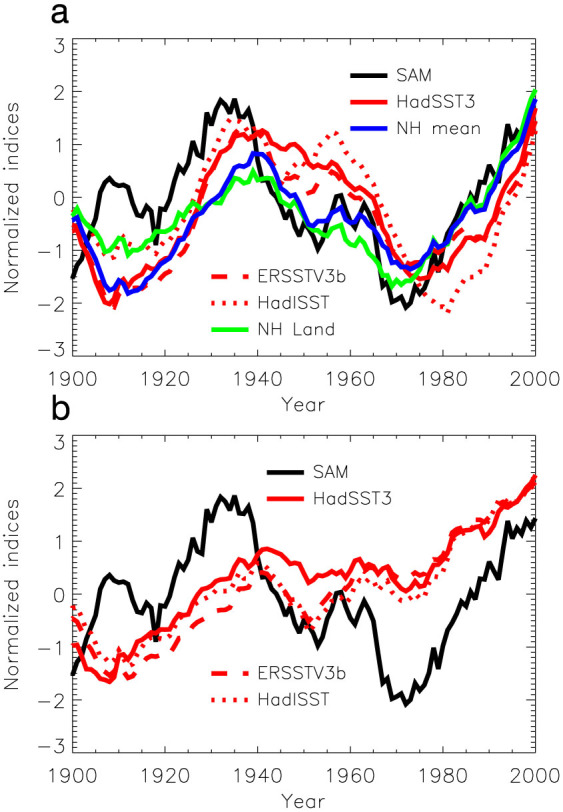
Coherent multi-decadal variability between the SAM and NH and tropical surface temperature. a, Time series of the Visbeck SAM index (black); AMO indices defined as average SSTs over the North Atlantic from the equator to 70°N from HadSST3 SST (red, solid), from ERSSTV3b (red, dashed), and from HadISST (red, dotted); averaged SAT over the NH land mass (green); NH mean surface temperature (blue). Time series are smoothed by applying the 11-year running mean, detrended, and normalized (see Fig. S5 for non-detrended time series). The correlation coefficients between the SAM index and the AMO indices and averaged SAT over the NH land mass after 1950 are 0.76 (p < 0.01) for HadSST3, 0.79 (p < 0.01) for ERSSTV3b, 0.50 (p < 0.01) for HadISST, 0.88 (p < 0.01) for averaged SAT over the NH land mass, and 0.93 (p < 0.01) for the NH mean surface temperature. b, The time series of averaged SST anomalies over 20°S–20°N (HadSST3: red, solid; ERSSTV3b: red, dashed; HadISST: red, dotted) and the SAM index (black). Time series of SST anomalies (relative to their means over the whole period) are smoothed by applying the 11-year running mean and normalized, but not detrended. Note that the result derived from the recently updated uninterpolated SST compilation, HadSST3, shows a clear cooling period between the 1940s and the 1970s. The correlation coefficients between the SAM index and the tropical surface temperatures after 1950 are 0.88 (p < 0.01) for HadSST3, 0.81 (p < 0.01) for ERSSTV3b, and 0.76 (p < 0.01) for HadISST. The significance levels of the correlations are determined by using the non-parametric random phase method^10^. This figure was plotted by using Interactive Data Language.

SAM, NH mean surface temperature, NH land SAT, and North Atlantic SSTs demonstrate coherent multi-decadal variability, especially after 1930 (Fig. 3a). A close examination of Fig. 3a indicates that the multi-decadal SAM variability corresponds well to major NH surface temperature fluctuations. The correlation coefficients between the SAM index and the AMO indices, averaged SAT over the NH land mass, and NH mean surface temperature after 1950 are 0.76 for HadSST3, 0.79 for ERSSTV3b, 0.50 for HadISST, 0.88 for the averaged SAT over the NH land mass, and 0.93 for the NH mean surface temperature; significance levels were determined to be less than 1% (these strong correlations are robust, as also supported by other available SAM and NH land SAT reconstructions, see Supplementary Fig. S5). Mechanisms responsible for NH multi-decadal SST and SAT variability have been extensively studied recently. Though the AMOC change^4^,^23^ was suggested to play a driving role, various studies have investigated mechanisms responsible for NH multidecadal temperature variability, including interplay between greenhouse gases and volcanic and anthropogenic aerosols forcings^24^,^25^,^26^ and intrinsic variability^27^.We note that both NH land SAT and SAM led the SST in phase transitions (Fig. 1e and Fig. 3a), in particular in the most recent decades when fidelity of the observational data was enhanced, indicating that NH land SAT and SAM phase transitions were not triggered by North Atlantic ocean circulation change.

Theoretical and modelling studies have shown that surface cooling or warming in the tropical region can lead to amplified air temperature changes in the upper tropical troposphere^28^, which can significantly alter the equator-to-pole temperature gradient in the upper troposphere and hence can strongly affect the SAM^19^. This motivated us to examine the relationship between the SAM variability and the tropical SST variability. We found that SAM index decreasing (increasing) generally corresponds to tropical surface cooling (warming) during the course of their multidecadal fluctuations after around 1940 (Fig. 3b), though some differences in fluctuation amplitudes and shorter time scale variability appear across the three datasets. In particular as shown in HadSST3, a recently updated SST dataset that has more realistic tropical SST variability^29^, the overall cooling trend between the 1940s and 1970s corresponds to the decreasing SAM index over the same period. The correlation coefficient between the SAM index and the detrended HadSST3 tropical surface temperature after 1950 is 0.93, and the correlation coefficient between the detrended HadSST3 tropical surface temperature and the detrended SO SST is 0.81 when the former leads the latter by 10 years; their significance levels were determined to be less than 1% using the non-parametric random phase method^10^.

To further elucidate how NH and tropical surface temperature changes can drive the SAM variability, we analysed vertical structures of global air temperature changes. The air temperature change analysis was conducted by using the atmospheric reanalysis datasets. After an evaluation of derived temperature trends from these datasets (for details see Supplementary Section VI), we elected to use the atmospheric reanalysis data, ERA-40^30^, for illustrating the involved mechanisms for the following reasons. First, the temperature trends in the tropical troposphere derived from this particular reanalysis are consistent with the observed tropical surface temperature changes, as determined by the basic nature of physics^28^. Second, ERA-40 data are available from September 1957 to August 2002, covering a cooling period (before roughly 1970) and then a warming period, permitting analysis of concurrent changes in global temperatures at different levels of the atmosphere for the periods with opposite surface temperature changes. Below, we highlight those temperature changes that are consistent with surface temperature changes and relevant to multi-decadal SAM variability.

**Figure 4:**
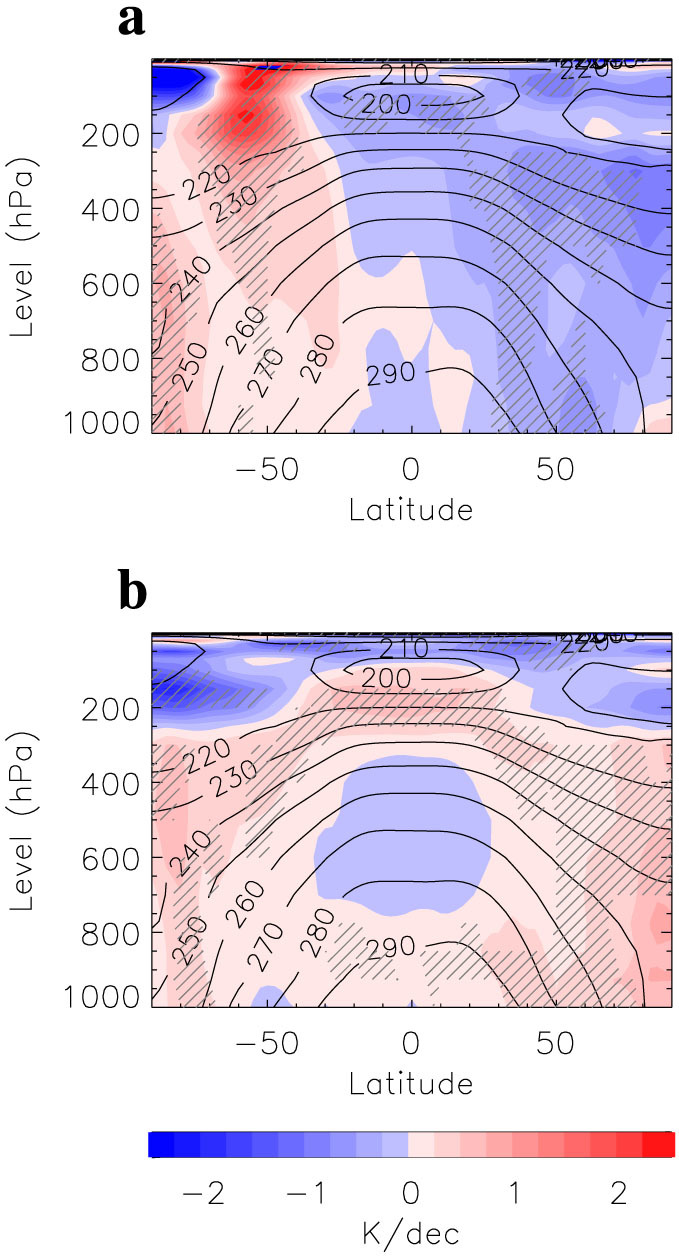
Influence of tropical tropospheric temperature change on SH equator-to-pole temperature gradient. a, b, Zonally averaged latitude-height cross sections of annual mean atmospheric temperature linear trends for the periods of 1958 to 1971 (a) and 1979 to 2001 (b). The trends are shown in colour, and the contour lines show the climatology mean for the period from 1958 to 2001. The significance levels of trends shown by hatched areas are less than 10%. This figure was plotted by using Interactive Data Language.

Significant, broad NH cooling is found within the zonal mean temperature of the lower troposphere and near the surface during 1958–71 (a surface cooling period in the tropical region) (Fig. 4a), indicating an upward extension of the NH land SAT and SST changes shown in Fig. 3a. In the middle troposphere cooling becomes larger and broader, while in the upper troposphere and the lower stratosphere changes are characterized by cooling at lower latitudes and relatively less cooling or even warming at high latitudes. Broad SH warming extends from the lower stratosphere to the surface, with warming much weaker at the surface. Overall, the most notable changes in atmospheric temperature during this period are surface cooling in the NH and tropics and amplified cooling in the tropical upper troposphere. This changed temperature pattern reduces the SH equator-to-pole temperature gradient in the lower stratosphere and the upper troposphere.

Between 1979 and 2001 (more reanalysis datasets have been developed for this satellite era since 1979, hence permitting an evaluation of consistency among these datasets for this particular period; see Supplementary Section VI for details), there was broad NH warming from the surface to the upper atmosphere, except for in the lower stratosphere, where cooling occurred (Fig. 4b). We highlight in particular two important features from the tropics to the SH middle and high latitudes: i) relatively large warming occurred, mainly in the middle troposphere at middle and high latitudes, and ii) significant cooling was exhibited above 350 hPa at the SH high latitudes, and a contrasting significant warming emerged in the tropical upper troposphere around 200 hPa.

This first feature suggests an increased static stability in SH mid-latitudes, which could push the eddy-driven jet poleward and strengthen westerly winds^31^,^32^. The second feature, characterized by amplified tropical-upper tropospheric warming due to increased latent heat release through enhanced moist convection^19^,^28^,^31^,^32^, indicates an increased equator-to-pole temperature gradient in the SH upper troposphere/lower stratosphere, leading to a strengthened upper-layer mid-latitude eastward wind^19^,^31^,^32^. This increased mid-latitude upper-layer wind is known to increase the eastward propagation speed of the mid-latitude eddies, which consequently pushes the wave-breaking region and the eddy-driven jet poleward^33^. Combined, the atmospheric temperature changes characterized by the two features described above can drive a strengthened SH westerly jet in recent decades.

Similarly, the reduced equator-to-pole temperature gradient in the SH lower stratosphere and upper troposphere, induced by amplified cooling in the tropical upper troposphere (Fig. 4a), can drive a weakened SH westerly jet in a cooling NH and tropical climate.

## Discussions

The finding above implies that NH warming may have played a triggering role in initiating multi-decadal SAM changes before the Antarctic ozone hole era^20^,^21^. It has been found that two major conditions are needed for the Antarctic ozone hole to form: i) the existence of polar stratospheric clouds which generally form at a temperature below −80°C, and ii) the existence of a large amount of ozone-depleting substances (mainly chlorofluorocarbons (CFCs) and Halons)^34^. Our results also imply that the strengthening of the SH westerly jet, initiated by the NH and tropical warming around 1970, could have enhanced the formation of polar stratospheric clouds and accelerated the destruction of Antarctic ozone through causing lower stratosphere cooling in the Antarctic winter and spring^34^.

By offering the evidence derived from the observational data and the observationally-constrained reanalysis data that SAM variability was initiated and was driven by the NH and tropical thermal state on multi-decadal time scales, a new hypothesis for the multi-decadal bipolar seesaw is advanced here: Cooling in the NH and tropics drove the weakening of the SH westerly jet, causing broad warming at SH high latitudes; conversely, NH and tropical warming initiated, and drove along with Antarctic ozone depletion, the strengthening of the SH westerly jet, causing broad cooling at SH high latitudes. Our analysis demonstrates that the independently observed NH land SAT, SST, and SH surface pressure data are dynamically consistent since 1950 when data quality dramatically improved. We thus propose that the observed multi-decadal bipolar seesaw has an atmospheric origin.

The finding in this study poses a new and great challenge to the current generation of climate models. In addition to reducing general uncertainties, a number of important and key relevant processes have to be identified and well resolved in the models to realistically simulate polar climate change and its interactive process with global climate, including the multi-decadal bipolar seesaw. For instance, when the westerly surface winds strengthen, sea salt aerosol concentration in the clouds would increase and, as a consequence, more short wave radiation will be reflected by clouds^14^, which has not been well treated in the state-of-the-art climate models. Note that there are various large uncertainties in reanalysis data sets, and future modeling work must be carried out for more complete understanding of the multi-decadal bipolar seesaw by using the coupled climate models that can appropriately resolve key relevant processes.

The results presented here indicate that atmospheric processes influence the recent multi-decadal variability of southern high-latitude surface temperatures. However, long-term sustained monitoring of AMOC in the North Atlantic, which is currently being undertaken (see http://www.noc.soton.ac.uk/rapidmoc/), is important for understanding the combined effects of atmospheric and oceanic circulation changes on multi-decadal climate variability.

## Methods

We used three long-term SST datasets: the Extended Reconstructed SST v3b since 1854^7^ (http://www.ncdc.noaa.gov/oa/climate/research/sst/ersstv3.php), HadISST since 1870^8^ (http://badc.nerc.ac.uk/data/hadisst/), and HadSST3 (uninterpolated) since 1870^9^ (http://www.metoffice.gov.uk/hadobs/hadsst3/). The long-term Visbeck SAM^12^ index was obtained from http://www.ifm-geomar.de/fileadmin/ifm-geomar/fuer_alle/PO/SAM/sam_annual.tab. The NH mean surface temperature and land surface temperature data^22^ were obtained from http://www.cru.uea.ac.uk/cru/data/temperature/. We also used ERA-40 reanalysis data^30^, which are available from http://data-portal.ecmwf.int/data/d/era40_moda/.

We used annual means calculated from monthly values and applied 11-year running averaging to smooth the time series in order to extract the multidecadal scale variability from the entire set of spectral variability signals in Figure 1 and Figure 3, and the significance levels of the smoothed time series correlations were determined by using the non-parametric random phase method^10^. Following the method in ref. 35, we used a t-test to determine the significance levels of the regressions and linear trends; the effective sample size was calculated as N(1 − r_1_r_2_)/(1 + r_1_r_2_), in which N is the sample size and r_1_ and r_2_ are the lag 1 autocorrelations of the two time series.

## Author Contributions

Z.W. and X.Z. conceived the study. Z.W. designed and conducted the analysis, and X.Z., Z.G., B.S., X.Y. and C.L. offered useful insights during this processes. Z.W. prepared the first draft, and X.Z., Z.G., B.S., X.Y. and C.L. joined in the writing of the paper. All authors reviewed the manuscript.

## Supplementary Material

Supplementary Information An atmospheric origin of the multi-decadal bipolar seesaw
