# Supplementary material for: An atmospheric origin of the multi-decadal bipolar seesaw
Source: Sci Rep. 2015 Mar 10;5:8909. doi: 10.1038/srep08909 (PMC4354075; doi:10.1038/srep08909)
Supplement: Supplementary Information — An atmospheric origin of the multi-decadal bipolar seesaw [file srep08909-s1.pdf]

Subject Area: Earth and Environmental Sciences

Correspondence and requests for materials should be addressed to Zhaomin Wang

Title: An atmospheric origin of the multi-decadal bipolar seesaw

Zhaomin Wang[1*], Xiangdong Zhang[2], Zhaoyong Guan[1], Bo Sun[3], Xin Yang[4] & Chengyan Liu[1]

[1]Polar Climate System and Global Change Lab, Nanjing University of Information Science and Technology (Nanjing Institute of Meteorology), Nanjing, China, 210044

[2]International Arctic Research Center and Department of Atmospheric Sciences, University of Alaska Fairbanks, 930 Koyukuk Dr., Fairbanks, Alaska 99775, USA

[3]Polar Research Institute of China, No. 451 Jinqiao Road, Pudong District, Shanghai, 200136, China

[4]British Antarctic Survey, High Cross, Madingley Road, Cambridge CB3 0ET, UK

[*] Corresponding author (wzm@nuist.edu.cn)

Supplementary Information

I. Heat budget analysis for the SO and its implications for AMOC change

Surface heat flux data from six datasets are used to analyze the heat balance of the SO. Two analysed heat fluxes used here, Large and Yeager[1] and OAFlux[2], are derived using satellite data and reanalysis data. The other four datasets, JRA-25 (http://jra.kishou.go.jp/JRA-25/index_en.html), CISL (http://dss.ucar.edu/datasets/ds093.2/), NCEP2 (http://nomad1.ncep.noaa.gov/pub/reanalysis-2/month/flx/), and ERA-40 (http://data-portal.ecmwf.int/data/d/era40_moda/), are results directly from numerical weather prediction (NWP) models. It has been noted that NWP fluxes contain systematic biases[3-5]. However, neither of the two analysed datasets (Larger and Yeager and OAFlux) are error-free either, as some surface variables are obtained from NWP analysis/reanalysis outputs, and satellite data could not be calibrated and validated if ground truth was very sparsely observed (this is particularly the case for the SO; for complete reviews on biases in surface flux data, see refs. 1 and 2). Thus, we have collected the flux data from these six datasets to increase the reliability of our results.

In ref. 6, the analysis of upper ocean heat content changes in the latitude band of 60°S-40°S for the top 700 m indicated a small warming trend between the 1980s and the 1990s (heat content increase of about $2\text{x}10^{22}$ J), and almost no change between the 1990s and the 2000s (see Fig. 6d of ref. 6). Using this result, as well as the collected surface heat flux data, we then examined whether the AMOC has played a major role in the SO heat balance since the 1980s. (This does not represent a very rigorous heat balance analysis, as we use the heat content change for the top 700 m only, and we assume much less dominant heat transport change at 60°S. This analysis can still offer useful insights, however, as i) refs. 7 and 8 show that substantial heat content changes have occurred in the upper ocean and are consistent with changes between 0 and 3000 m; and ii) the mean heat transport at 60°S is indeed much smaller than that at 40°S[9].) If the SO surface cooling after around 1980 (see Fig. 1 in the

letter) was predominantly caused by the AMOC strengthening that moved heat away from the SO region, we would expect this AMOC strengthening to be qualitatively consistent with the changes in the heat content and surface net heat input into the SO; i.e., there should be a considerable increase in the net surface heat input into the SO, to balance the small warming trend.

Fig. S1a shows the area-averaged anomalies of net surface heat fluxes over 60°S-40°S for the six datasets (black solid: Large and Yeager; red solid: OAFlux; blue solid: JRA-25; black dashed: CISL; red dashed: NCEP2; blue dashed: ERA-40) after 1980. We first discuss the results from Large and Yeager and OAFlux, the two analysed heat flux datasets that are not directly from NWP models. Results from these two datasets show substantially reduced net heat input into the SO before 1995, but increased heat input afterwards. Changes in the net surface heat flux between 1980 and 1995 are large. For example, the linear trend in the Large and Yeager heat flux integrated over 60°S-40°S between 1980 and 1995 is -6.7 W/m^2 per decade, which must be balanced by an increase of 0.38 PW (1 PW = 10^{15} W) per decade in poleward heat transport at 40°S for zero heat content change in the latitude band of 60°S-40°S. Such a flux is alternatively equivalent to a heat content decrease of 6.0×10^{22} J for a zero meridional heat transport change; a very large AMOC weakening is required if the AMOC is dominant in the SO heat balance. Despite the increase during the latter half of this period, the overall trends during the whole period are negative, particularly for Large and Yeager (-2.6 W/m^2/dec and -1.2 W/m^2/dec for Large and Yeager and OAFlux, respectively). Clearly, these surface heat flux changes indicate a decrease in surface heat input into the SO, thus requiring a net oceanic heat transport towards this region. If the AMOC was the dominant process in the poleward heat transport at 40°S, a weakening of the AMOC should occur. Clearly, this cannot be reconciled with the observed large northern warming trend during this period, if the

AMOC was a primary contributor to this warming (see Fig. 1 in the letter). The heat balance implied by these data suggests, therefore, that it is difficult to invoke the AMOC to explain both the northern and the southern multi-decadal SST variability.

The other four datasets used are derived from NWP models and hence have larger possible uncertainties. However, for the period before the mid-1990s, they generally agree with the above results. Only over the whole period are the linear trends not consistent (-2.0 $W/m^2/dec$, 0.0 $W/m^2/dec$, -0.8 $W/m^2/dec$, and 1.1 $W/m^2/dec$ for JRA-25, CISL, NCEP2, and ERA-40, respectively); this inconsistency is caused mainly by large discrepancies after the mid-1990s. The ensemble mean of the anomalies of these six datasets (Fig. S1b) exhibits a consistent downward trend, suggesting that there may have been a slight weakening of the AMOC over recent decades, consistent with the result of an existing AMOC observational study[10].

Note that currently available surface heat flux data may still need to be improved, and that the results presented here may not be very robust. However, the results obtained from analyzing these six datasets are consistent with the finding that there was a negative (cooling) aerosol climate feedback induced by the increase in westerly winds since the 1970s, reflecting more summer solar insolation[11]; also, the almost simultaneous variations of the observed northern and SO SST changes (see Fig. 1 in the letter) suggest that there may be an atmospheric origin of the multi-decadal bipolar seesaw.

II. Uncertainties in the SST

Historical SST observations for the SO are particularly sparse. Here we show the number of SST observations for our two regions of interest (the southern region defined as between 70°S and 50°S of the SO, and the northern region defined as between 50°N and 70°N of the North Atlantic), from 1880 to 1980, in Fig. S2 (after 1980, the number of observations increased

considerably in the two regions). Data are from ICOADS (International Comprehensive Ocean-Atmosphere Data Set; http://www.esrl.noaa.gov/psd/data/gridded/data.coads.2deg.html). The number of SST observations in the northern region (black) is much larger than that in the southern region (red) after about 1900. For the northern region, there were two dramatic drops—between 1915 and 1920 (the First World War period) and around 1940. There were almost no observations for the southern region in the 1930s and during the Second World War, even in the austral summer. Although there were some observations before 1920, spatial coverage was very much limited to the northern part of the southern region in the South Pacific sector (see Fig. 3 of ref. 12), and much less uniformly distributed than afterwards. For the southern region, there were obvious increases in the number of summer season observations after about 1950. However, the winter SST observations in this region continued to be very sparse until about 1980.

In addition to a lack of observations, SST biases could also result from instrument changes. For example, the sudden drop in global SST in 1945 was identified as having been caused by the change from engine room intake measurements (US ships) to uninsulated bucket measurements (UK ships)[13]. This also likely contributed to the drop in northern SST around 1945 shown in Fig. 1e and 1f in the letter.

III. Robustness of the bipolar seesaw since the 1940s

Since most ship-board observational data were only available for summer seasons in the SO before about 1980, ERSSTV3b, HadISST, and HadSST3 annual mean data could be biased in this region. Although seasonal bias could be removed by correcting for a seasonal cycle in SST[14], there remains the possibility that apparent recent annual cooling could have resulted from the increased use of float data during winter. We thus plotted a figure (Fig. S3), similar

to Figs. 1e and 1f from the letter, using SST data from summer seasons (July, August, and September for the northern region; January, February, and March for the southern region) only. Within these summer-only data, recent cooling in the SO remains a robust result for the SST compilations used, so the bipolar seesaw pattern remains a robust feature since the 1940s.

After the 1940s, the bipolar seesaw pattern in the observed SST is consistent with the results derived from independent polar SAT data[15], when SST and polar SAT data are more reliable, and it is also consistent with upper layer heat content change in the SH oceans[6].

Surface regional multi-decadal variability patterns have also been shown to be consistent with the observed subsurface ocean temperature change for recent decades, after applying a bias correction to XBT (expendable bathythermograph) fall rate[16]. Furthermore, the surface cooling in the SO since the 1970s is consistent with an Antarctic temperature reconstruction[17] improved from Steig et al. (2009)[18]. While ref. 19 questioned the robustness of the bipolar seesaw before the 1940s, the results from this study and the studies mentioned above demonstrate that the bipolar seesaw pattern is a robust feature at least since the 1940s, after which instrumental data quality has dramatically improved.

IV. Responses of the SO SST to tropical Pacific SST anomalies

From year to year, large-scale climate variability at southern high latitudes is also influenced by the remote response to the tropical Pacific SST anomalies associated with the El Niño-Southern Oscillation (ENSO) phenomenon. The SH atmospheric response to ENSO exhibits wavelike patterns, leading to a zonally asymmetric response of the SST in the SO. On decadal and multi-decadal scales, similar to ENSO events but with broader meridional and zonal scales for tropical anomalies, the Inter-decadal Pacific Oscillation (IPO) has been

identified as a Pacific-wide phenomenon[20]. In contrast to the ENSO phenomenon, IPO structure is more symmetric around the equator and much less variable in the easternmost Pacific. There is remarkably large variability in the extratropics associated with IPO[20]. For a more complete analysis of the multi-decadal SST variability in the SO, the response of the SO SST to tropical Pacific SST anomalies must also be investigated on multi-decadal scales.

We calculated the regression of the annual mean SSTs on the IPO index derived by ref. 20 (color portions of Fig. S4a and Fig. S4b). In these two panels, there are pronounced, zonally asymmetric SST changes in response to decadal-scale tropical SST anomalies. The results for both SST datasets show consistent warming (corresponding to positive IPO; i.e., in response to a positive, tropical SST anomaly) with significance levels of less than 5 % in the eastern Pacific sector of the SO, and consistent (though less statistically significant) cooling between roughly the Prime Meridian and 90°E. In the latter region, the cooling is in large contrast to the warming obtained from the annual means of monthly regressions of the SST on NINO3.4 (average SST over 5°S-5°N and 120°W-170°W; obtained using monthly SST from 1981 to 2005; negative contours are dashed and the zero contour is highlighted in bold, with a contour interval of 0.2) for both ERSSTV3b and HadISST.

Fig. S4c shows the IPO index (thick black), the Visbeck SAM index[21] (thick red), and the selected SST time series for which there is a large correlation coefficient (60°S, 125°W; thin black). Note that the correlation between the SAM and the IPO indices is low (correlation coefficient of 0.26). Before 1920, the SST at 60°S, 125°W is poorly correlated with the IPO index, presumably a consequence of large uncertainties in the SO SST before that time.

The multi-decadal-scale SO SST-SAM/IPO relationships established here and in the letter can be used to explain the more detailed spatial distributions of the SO SST changes in the

past several decades, as shown in Fig. 1 of the letter. The broad, quasi-zonal cooling at southern high latitudes has been caused mainly by the increased atmospheric forcing associated with the annular mode, while the changes in tropical SST forcing modulated SST changes regionally. For example, for the SST trend between 1980 and 2009 shown in Fig. 1d for HadISST, the recently increased westerly forcing is presumed to cause warming to the west of Drake Passage, as suggested by Fig. 2b. However, the drop in IPO index since 1982 tends to cause cooling in this area, cancelling the SST change induced by strengthened westerlies. Also, the SAM-induced cooling around 90°E was cancelled by the warming effect of the tropical SST forcing, even leading to a small net warming.

V. Comparisons between three reconstructed SAM indices and land SATs from two datasets

After the reconstruction of the Visbeck SAM index[21], two more long-term (1905-2005) SAM indices (Jones and Widmann SAM index, Fogt SAM index) were reconstructed[22]. The first principle component of extratropical sea level pressure was used as a predictand in the Jones and Widmann reconstruction, and the method for deriving the Marshall SAM index[23] was further used to derive the Fogt SAM index. Detailed descriptions of these two reconstructions are given in ref. 22, and comparisons of these two SAM indices along with the Visbeck SAM index[21] were also conducted, season by season, as shown in ref. 22. In Fig. S5 we show that these three indices are generally consistent on multi-decadal scales. Also, average land SATs over the NH from CRU (http://www.cru.uea.ac.uk/cru /data/temperature/) and GISS (http://data.giss.nasa.gov/gistemp/) are generally consistent.

VI. Comparisons of temperature trends among reanalysis datasets and direct measurements

Two further atmospheric reanalysis—NCEP1 (National Centers for Environmental Prediction Phase 1) (http://www.esrl.noaa.gov/psd/data/gridded/data.ncep.reanalysis .pressure.html) and 20thC_Rean (20th century reanalysis) (http://www.esrl.noaa.gov/psd/data/gridded/data.20thC_ReanV2.pressure.mm.html)—cover two long periods of 1948-2011 and 1871-2010, respectively. Thus, the analysed temperature trends during 1958-1971 in ERA-40 can be compared with the results derived from these two datasets. Similar to the trends derived from ERA-40, there is also broad cooling in the NH and in the tropical upper troposphere in these two datasets (Fig. S6), though the trends are not statistically significant (significance levels greater than 10 %) in many regions, due to short time periods. Large discrepancies exist at southern high latitudes, where there occurs large warming in NCEP1 and ERA-40, but large cooling in the upper troposphere and stratosphere in 20thC_Rean.

For the period of 1979-2001, more additional reanalysis datasets are available. Beyond the two datasets above, here we analysed 1979-2001 temperature trends within CFSR (NCEP Climate Forecast System Reanalysis) (http://dss.ucar.edu/datasets/ds093.2/) and ERA-Interim (http://data-portal.ecmwf.int/data/d/interim) (Fig. S7). In these four datasets, there is broad and statistically significant warming in the NH, similar to the result from ERA-40. In the tropical upper troposphere, except for NCEP1, there are general warming trends consistent with the results of ERA-40, though the trend in ERA-Interim is relatively small. In these four datasets, over the Antarctic region there are consistent cooling trends above the middle troposphere and significantly less consistent trends in the lower troposphere.

Despite large discrepancies for the temperature trends derived from these reanalysis data, the tropical upper troposphere has generally exhibited greater warming than the surface since 1979. As analyzed in ref. 24 (and references therein), however, the tropical upper troposphere has warmed less than the surface, or has even become cooler in some radiosonde and satellite datasets since 1979, contradicting the results derived from these reanalysis data and in coupled climate models[24]. Several studies have suggested that these cooling biases may indeed represent residual inhomogeneities in these observations, hampering the detection of multi-decadal variability using these observational data (see references in ref. 24). It has been observed that the tropical surface has warmed since 1979 (Fig. 4a and ref. 24), so this decadal-scale warming should enhance moist convection and lead to larger upper tropospheric warming according to the very basic nature of the involved physics. The greater warming in the tropical upper troposphere is further supported by other independent evidence, such as increases in tropospheric water vapour and tropopause height (see references in ref. 24).

This same mechanism should also have been operating at earlier times, with surface cooling (e.g., between the 1940s and 1970s as shown in Fig. 4a) leading to greater cooling in the tropical upper troposphere than at the surface, through reduced moist convection. Though there are some discrepancies with respect to trends in surface temperature during the 1940s and 1970s in the datasets shown in Fig. 4c, the result of HadSST3 is more plausible. This is because i) this recently updated version of the Hadley Centre SST dataset can capture more realistic SST changes than HadISST and ERSSTV3b in the tropical region[25]; ii) the SST change between the 1940s and 1970s in the tropical region is consistent with those changes derived from average SST over the North Atlantic and average land SAT over the NH; and iii)

SST cooling and implied amplified cooling in the tropical upper troposphere are dynamically consistent with the weakening of the SH westerly winds from the 1940s to 1970s.

In summary, the results obtained from the above comparisons and from previous theoretical and modelling studies (e.g., ref. 24 and references therein) strongly support the results from analyzing ERA-40 atmospheric temperature data as shown in the letter: when there is broad NH cooling, there is both surface cooling and amplified upper troposphere cooling in the tropical region; similarly, there is larger tropical upper troposphere warming induced by surface warming when there is broad NH warming. These temperature changes can modify the meridional temperature gradient of the upper layer that governs the changes of the SH westerly jet.

Figure captions

Fig. S1: a) Area-averaged anomalies of net surface heat input into the SO within the latitude band of 60^oS-40^oS since 1980, derived from the six surface heat flux datasets (Large and Yeager: solid black; OAFlux: solid red; JRA-25: solid blue; CISL: dashed black; NCEP2: dashed red; ERA-40: dashed blue). b) The ensemble mean (thick line) and the mean plus and minus one standard deviation (thin lines) of the area-averaged anomalies of net surface heat input into the Southern Ocean within the latitude band of 60^oS-40^oS since 1980, derived from the six heat flux datasets. This figure was plotted by using Interactive Data Language.

Fig. S2: Time series of monthly totals of SST observations (in 10^4) for the northern region (Atlantic sector of 50^oN-70^oN) (black) and for the southern region (70^oS-50^oS) (red), between 1880 and 1980. This figure was plotted by using Interactive Data Language.

Fig. S3: Time series of northern and southern (or SO SST) anomalies, derived from summer (July, August, and September for the northern SST; January, February, and March for the

southern or SO SST) data only. Time series are smoothed by applying the 11-year running mean (ERSSTV3b: black, HadISST: blue, HadSST3: red; solid lines are for southern or SO SSTs averaged over 70°S-50°S, and dashed lines are for northern SSTs averaged over the Atlantic sector of 50°N-70°N.) Correlation coefficients between the SO SST and northern SST are -0.33 (p=0.01) for ERSSTV3b, -0.94 (p<0.01) for HadISST, and -0.26 (p=0.05) for HadSST3. This figure was plotted by using Interactive Data Language.

Fig. S4: Linear regressions of a) ERSSTV3b SST and b) HadISST SST on IPO index (color portion). The significance levels at hatched areas are less than 5 %, and the annual means of the monthly regressions of the SST on ENSO indices are shown as contour lines (negative contours are dashed and the zero contour is highlighted in bold; contour interval: 0.2). The time series of IPO index (thick black), Visbeck SAM index (thick red), and selected SST time series at 60°S, 125°W from HadISST (thin black) are plotted in c). This figure was plotted by using Interactive Data Language.

Fig. S5: Time series of the Visbeck SAM index (thick black solid), the Jones and Widmann SAM index (thick black dotted), and the Fogt SAM index (thick black dashed). The two averaged land SATs over NH are from CRU (solid green; http://www.cru.uea.ac.uk/cru /data/temperature/) and GISS (dashed green; http://data.giss.nasa.gov/gistemp/). As in Fig. 3 from the letter, three AMO indices (in red—HadSST3: solid; ERSSTV3b: dashed; HadISST: dotted) are also shown. The time series are smoothed by applying the 11-year running mean and normalized, but not detrended. The correlation coefficients are 0.87 (p<0.01) between the Visbeck SAM index and Jones and Widmann SAM index and 0.93 (p<0.01) between the Visbeck SAM index and Fogt SAM index after 1950. The correlation coefficient is 0.88 (p<0.01) between the GISS land SAT and CRU land SAT after 1950. This figure was plotted by using Interactive Data Language.

Fig. S6: Linear trends in atmospheric temperature over 1958-1971 for a) NCEP1 and b)

20thC_Rean. Significance levels at hatched areas are less than 10 %. This figure was plotted by using Interactive Data Language.

Fig. S7: Linear trends in atmospheric temperature over 1979-2001 for a) NCEP1; b) 20thC_Rean; c) CFSR; and d) ERA-Interim. Significance levels at hatched areas are less than 10 %. This figure was plotted by using Interactive Data Language.

References

1. Large, W. G. & Yeager, S. G. The global climatology of an interannually varying air-sea flux dataset, *Clim. Dynam.*, **33**, 341-364 (2009).

2. Yu, L., & Weller, R. A. Objectively analyzed air-sea heat fluxes for the global ice-free oceans (1981-2005). *Bull. Amer. Met. Soc.*, **88**, 527-539 (2007).

3. Moyer, K. A. & Weller, R. A. Observations of surface forcing from the subduction experiment: A comparison with global model products and climatological datasets, *J. Climate*, **10**, 2725-2742 (1997).

4. Josey, S. A. A Comparison of ECMWF, NCEPNCAR, and SOC surface heat fluxes with moored buoy measurements in the subduction region of the Northeast Atlantic, *J. Climate*, **14**, 1780-1789 (2001).

5. Smith, S. R., Legler, D. M. & Verzone, K. V. Quantifying uncertainties in NCEP reanalyses using high-quality research vessel observations, *J. Climate*, **14**, 4062-4072 (2001).

6. Gille, S. Decadal-scale temperature trends in the Southern Hemisphere ocean, *J. Climate*, **21**, 4749-4765, DOI: 10.1175/2008JCLI2131.1 (2008).

7. Levitus, S., Antonov, J. L., Boyer, T. P. & Stephens, C. Warming of the world ocean, *Science*, **287**, 2225–2229 (2000).

8. Levitus, S., Antonov, J. & Boyer, T. Warming of the world ocean, 1955-2003, *Geophys. Res. Lett.*, **32,** L02, 604, doi:10.1029/2004GL021,592 (2005).

9. Trenberth, K. E. & Caron, J. M. Estimates of meridional atmosphere and ocean heat transports, *J. Climate*, **14**, 3433–3443 (2001).

10. Longworth, H. R., Bryden, H. L. & Baringer, M. O. Historical variability in Atlantic meridional baroclinic transport at 26.5°N from boundary dynamic height observations, *Deep-Sea Res. Part II*, **58**, 1754-1767 (2011).

11. Korhonen, H., Carslaw, K. S., Forster, P. M., Mikkonen, S., Gordon, N. D., & Kokkola, H. Aerosol climate feedback due to decadal increases in Southern Hemisphere wind speeds, *Geophys. Res. Lett.* **37**, L02805, doi:10.1029/2009GL041320 (2010).

12. Deser, C., Alexander, M. A., Xie, S.-P. & Phillips, A. S. Sea surface temperature variability: Patterns and mechanisms. *Annu. Rev. Mar. Sci.* **2**, 115–143 (2010).

13. Thompson, D. W. J., Kennedy, J. J., Wallace, J. M. & Jones, P. D. A large discontinuity in the mid-twentieth century in observed global-mean surface temperature. *Nature* **453**, 646–649 (2008).

14. Gouretski, V. & Koltermann, K. P. How much is the ocean really warming? *Geophys. Res. Lett.* **34** , L01610, doi:10.1029/2006GL027834 (2007).

15. Chylek, P., Folland, C. K., Lesins, G. & Dubey, M. K. Twentieth century bipolar seesaw of the Arctic and Antarctic surface air temperatures. *Geophys. Res. Lett*. **37**, L08,703, doi:10.1029/2010GL042,793 (2010).

16. Carson, M. & Harrison, D. E. Regional interdecadal variability in bias-corrected ocean temperature data. *J. Climate* **23**, 2847–2855 (2010).

17. O'Donnell, R., Lewis, N., McIntyre, S. & Condon, J. Improved methods for PCA-based reconstructions: Case study using the Steig et al. (2009) Antarctic temperature reconstruction, *J. Clim.* **24**, 2099–2115 (2011).

18. Steig, E. J., Schneider, D. P., Rutherford, S. D., Mann, M. E., Comiso, J. C. & Shindell, D. T. Warming of the Antarctic ice-sheet surface since the 1957 International Geophysical Year. *Nature* **457**, 459–462 (2009).

19. Schneider, D. P. & Noone, D. C. Is a bipolar seesaw consistent with observed Antarctic climate variability and trends?, *Geophys. Res. Lett.* **39**, L06704, doi:10.1029/2011GL050826 (2012).

20. Parker, D. *et al*. Decadal to multidecadal variability and the climate change background. *J. Geophys. Res.* **112**, doi:10.1029/2007JD008,411 (2007).

21. Visbeck, M. A station-based southern annular mode index from 1884 to 2005. *J. Climate* **22**, 940-950 (2009).

22. Jones, J. M. *et al*. Historical SAM variability. Part I: Century-length seasonal reconstructions. *J. Climate* **22**, 5319–5345 (2009).

23. Marshall, G. J. Trends in the Southern Annular Mode from observations and reanalyses. *J. Climate* **16**, 4134–4143 (2003).

24. Santer, B.D. *et al.* Amplification of surface temperature trends and variability in the tropical atmosphere. *Science* **309**, 1551-1556 (2005).

25. Tokinaga, H., Xie, S.-P., Deser, C., Kosaka, Y. & Okumura, Y. M. Slowdown of the Walker circulation driven by tropical Indo-Pacific warming, *Nature* **491**, 439-444 (2012).

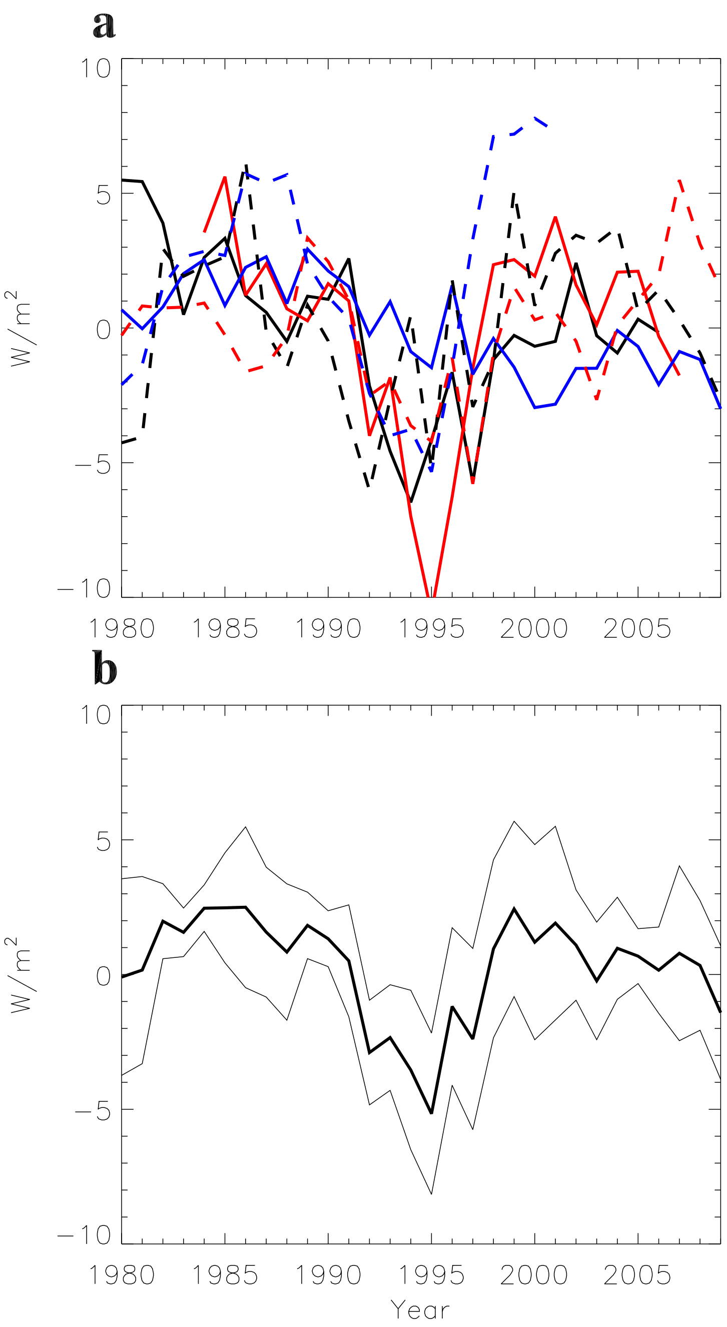
a
10
5
0
−5
−10
W/m2
1980
1985
1990
1995
2000
2005
b
10
5
0
−5
−10
W/m2
1980
1985
1990
1995
2000
2005
Year

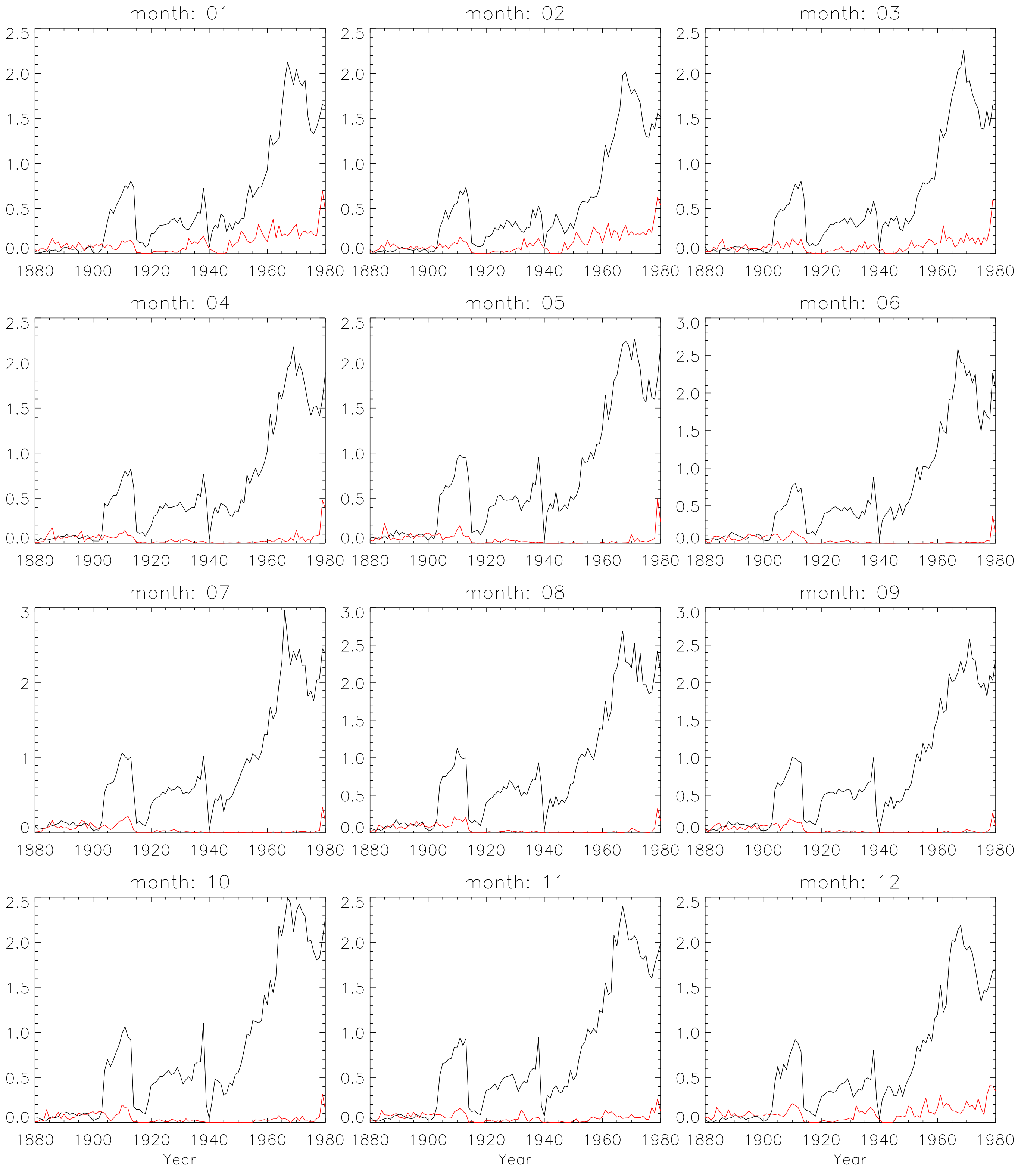
month: 01
month: 02
month: 03
month: 04
month: 05
month: 06
month: 07
month: 08
month: 09
month: 10
month: 11
month: 12
Year
Year
Year

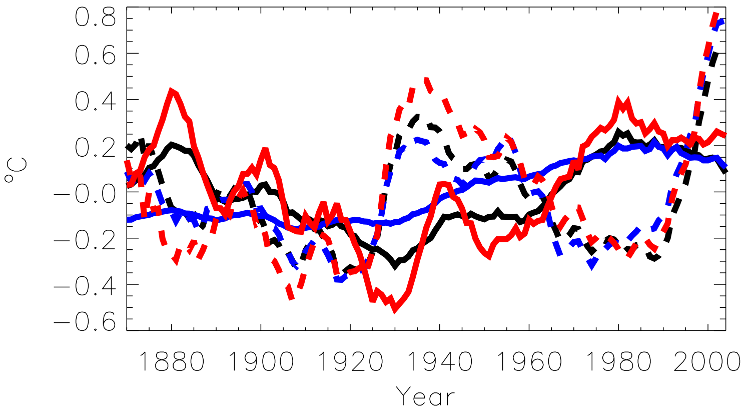
0.8
0.6
0.4
0.2
−0.0
−0.2
−0.4
−0.6
°C
1880
1900
1920
1940
1960
1980
2000
Year

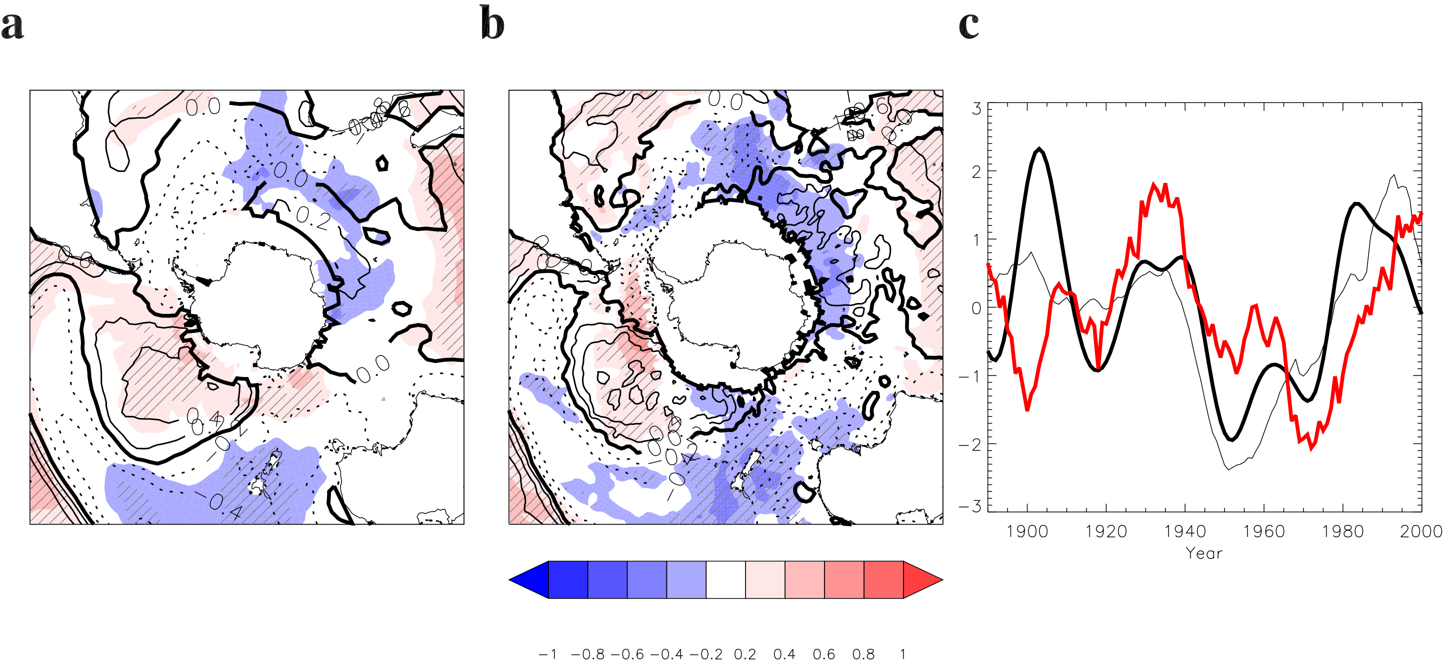

a
b
c
1900
1920
1940
1960
1980
2000
Year
−1 −0.8 −0.6 −0.4 −0.2 0.2 0.4 0.6 0.8 1

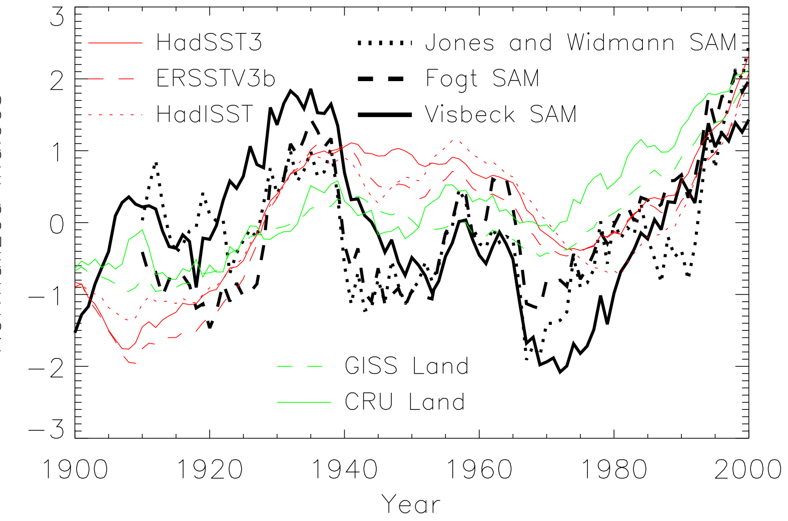

HadSST3
ERSSTV3b
HadISST
Jones and Widmann SAM
Fogt SAM
Visbeck SAM
GISS Land
CRU Land
3
2
1
0
−1
−2
−3
1900
1920
1940
1960
1980
2000
Year

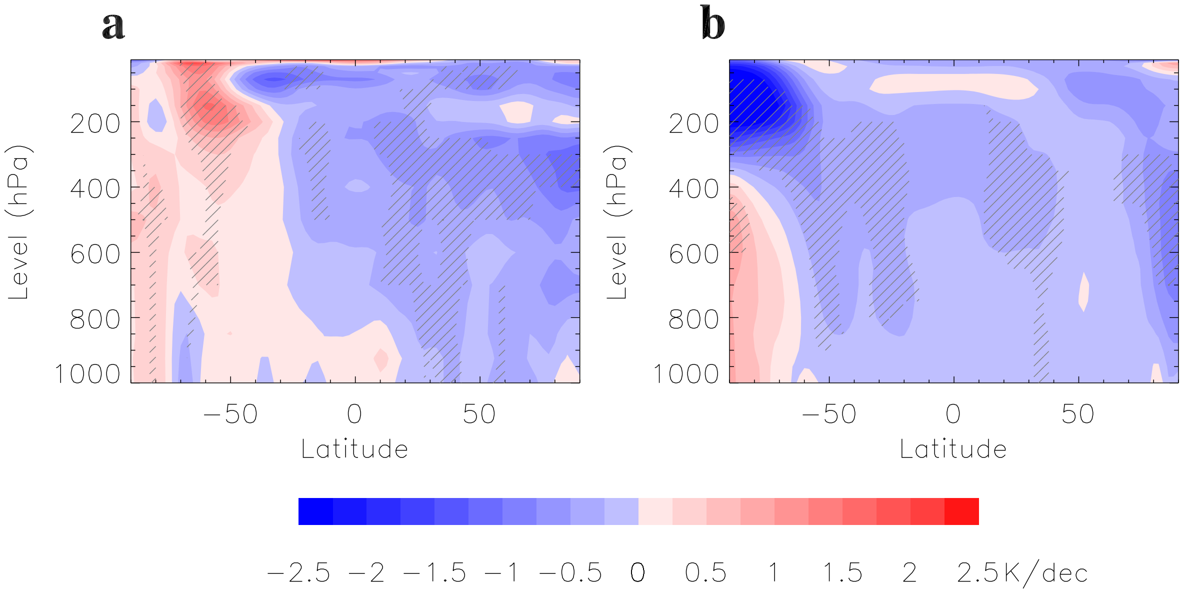

a
b
200
400
600
800
1000
Level (hPa)
−50
0
50
Latitude
−2.5 −2 −1.5 −1 −0.5 0 0.5 1 1.5 2 2.5K/dec

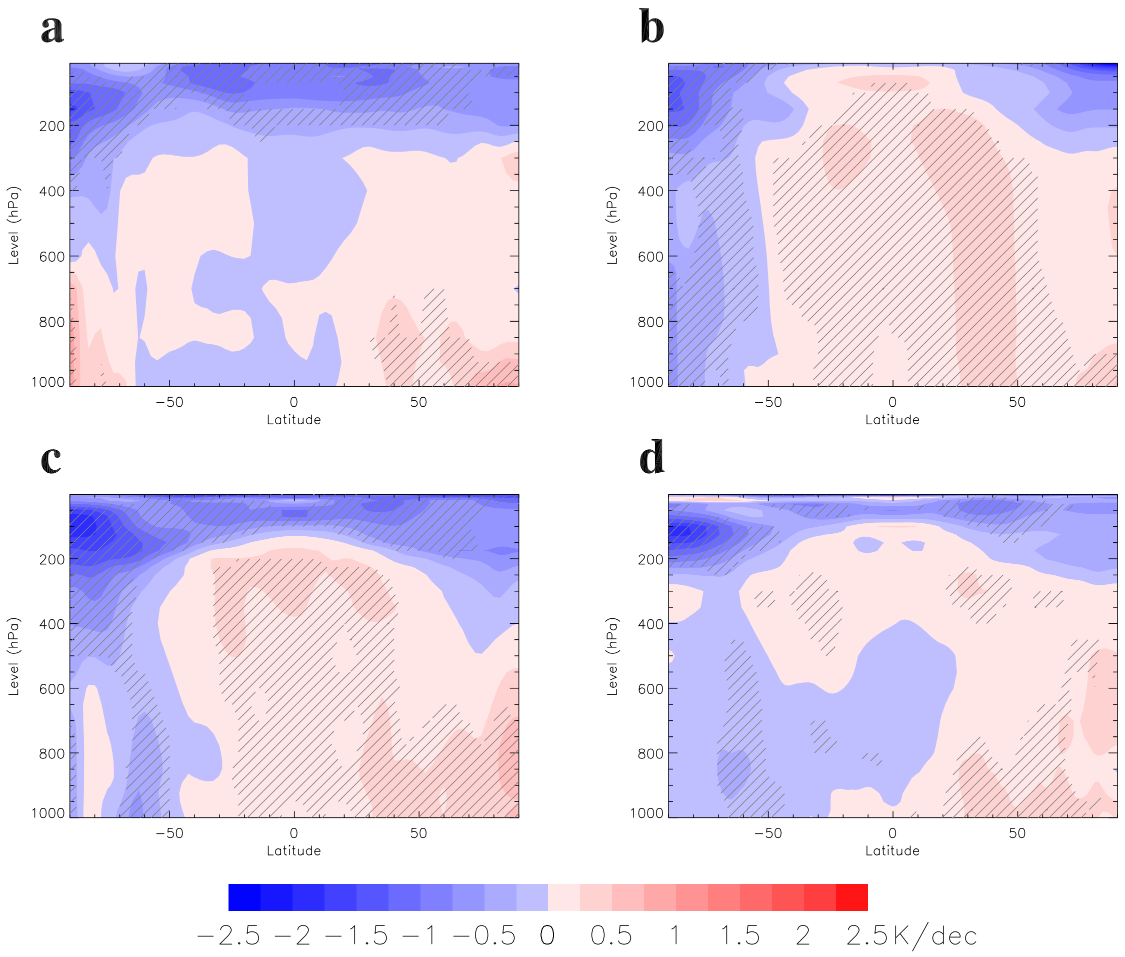

a
b
c
d
Level (hPa)
200
400
600
800
1000
−50
0
50
Latitude
−2.5 −2 −1.5 −1 −0.5 0 0.5 1 1.5 2 2.5K/dec
